# Influence of Oral and Gut Microbiota in the Health of Menopausal Women

**DOI:** 10.3389/fmicb.2017.01884

**Published:** 2017-09-28

**Authors:** Angélica T. Vieira, Paula M. Castelo, Daniel A. Ribeiro, Caroline M. Ferreira

**Affiliations:** ^1^Department of Biochemistry and Immunology, Institute of Biological Sciences, Federal University of Minas Gerais, Belo Horizonte, Brazil; ^2^Department of Pharmaceutics Sciences, Institute of Environmental, Chemical and Pharmaceutical Sciences, Universidade Federal de São Paulo, Diadema, Brazil; ^3^Pathology Graduate Program, Universidade Federal de São Paulo, São Paulo, Brazil; ^4^Department of Biosciences, Universidade Federal de São Paulo, Santos, Brazil

**Keywords:** saliva, oral health, mouth diseases, gut microbiota, estrogen, menopause

## Abstract

Sex differences in gut microbiota are acknowledged, and evidence suggests that gut microbiota may have a role in higher incidence and/or severity of autoimmune diseases in females. Additionally, it has been suggested that oral, vaginal, and gut microbiota composition can be regulated by estrogen levels. The association of vaginal microbiota with vulvovaginal atrophy at menopause is well described in the literature. However, the relevance of oral and gut microbiota modulation in the immune system during estrogen deficiency and its effect on inflammatory diseases is not well explored. Estrogen deficiency is a condition that occurs in menopausal women, and it can last approximately 30 years of a woman’s life. The purpose of this mini- review is to highlight the importance of alterations in the oral and gut microbiota during estrogen deficiency and their effect on oral and inflammatory diseases that are associated with menopause. Considering that hormone replacement therapy is not always recommended or sufficient to prevent or treat menopause-related disease, we will also discuss the use of probiotics and prebiotics as an option for the prevention or treatment of these diseases.

## Introduction

We harbor trillions of microorganisms that associate with specific tissues and are termed microbiota. This rich community of microorganisms, mostly bacteria, has co-evolved in a symbiotic relationship with humans in such a way that it is now essential for several physiological functions and controls many aspects of host physiology (Backhed et al., 2005; Backhed, 2012; Grover and Kashyap, 2014).

One of the factors that plays a pivotal role in microbiota modulation, although broadly understudied in current research, is the change in female sexual hormones throughout life. Two phases occur in a woman’s life that are characterized by several physiological, metabolic and immunological changes: menarche, or the first menstruation of a woman, which occurs during adolescence between 10 and 15 years of age (Hoffmann et al., 2004), and menopause, which occurs between age 45 and 55 and includes the cessation of menstrual periods and loss of the reproductive function of the ovaries (Brotman et al., 2014). In fact, estrogen and the microbiota of a woman’s body tend to be investigated more extensively during the woman’s reproductive years than during menopause or the phase of estrogen decline. One exception is the vaginal microbiota, which has been widely investigated during menopause. Here, we consider menopause or the menopausal phase, including perimenopause (before menopause), menopause and postmenopause (after menopause).

Considering that menopause can last for approximately 30 years of a woman’s life (Brotman et al., 2014), the purpose of this mini-review is to highlight the importance of alterations in the oral and gut microbiota during estrogen deficiency and determine their relevance in oral infections and inflammatory diseases that are associated with menopause.

## The Interaction Between Oral Microbiota and Female Sex Hormones

The oral cavity (mouth) is composed of several distinct microbial habitats, including the lips, the teeth, the gingival sulcus, the tongue, the cheeks, the palate and the tonsils, which are colonized by hundreds of different bacterial, viral, and fungal species (Dewhirst et al., 2010; Yost et al., 2015). The microbial communities associated with these structures are in symbiosis with the host (Sanz et al., 2017). However, in the presence of stressors that can perturb this homeostasis, several oral infectious diseases may appear, including dental caries and periodontitis (Almstahl et al., 2010; Dewhirst et al., 2010). Many of these disease are recognized to be caused by the consortia of organisms in a biofilm rather than a single pathogen (Jenkinson and Lamont, 2005). In addition, poor oral health and oral diseases may be associated with many systemic diseases (Seymour et al., 2007), such as cardiovascular diseases (Joshipura et al., 1996; Montebugnoli et al., 2004; Belenguer et al., 2006), stroke (Joshipura et al., 2003), preterm birth (Offenbacher et al., 1998), diabetes (Genco et al., 2005), and pneumonia (Awano et al., 2008).

In healthy individuals, the microorganisms found in the mouth with the largest representation include *Streptococcus*, *Actinomyces*, *Veillonella*, *Fusobacterium*, *Porphyromonas*, *Prevotella*, *Treponema*, *Neisseria*, *Haemophilus*, *Eubacteria*, *Lactobacterium*, *Capnocytophaga*, *Eikenella*, *Leptotrichia*, *Peptostreptococcus*, *Staphylococcus*, and *Propionibacterium* (Jenkinson and Lamont, 2005; Liu et al., 2012). The behavior of these organisms can be very dynamic and adapt to a wide range of environments and interactions with other microbial species while aggregated in biofilms over the oral surfaces.

Estrogen receptor-beta has been detected in the oral mucosa and salivary glands (Valimaa et al., 2004), and some evidence shows age-related hormonal changes in the exfoliated normal buccal mucosa of women (Donald et al., 2013). Moreover, the vaginal and buccal epithelia share some microscopic similarities. As observed by Thompson et al. (2001), the patterns of surface keratinization and the distribution and appearance of the lipid lamellae in the intercellular spaces were similar between vaginal and buccal epithelial samples of postmenopausal women. Therefore, given that many menopausal women also suffer from oral discomforts in addition to climacteric symptoms (Meurman et al., 2009), an understanding of the impact of female sex hormones on the characteristics of the oral microbiota may be clinically relevant, especially during menopause. Some of the main complaints from women in menopause include dry mouth and tooth loss, and the existing data have focused on the salivary microbial composition and the microbiota characteristics of the gingival sulcus. Therefore, this review will explore the main findings of the relationship between the oral microbiota and menopause in saliva and periodontal support.

### Saliva

Saliva plays an important role in the maintenance of oral health integrity and the protection against dental caries and other oral diseases (Marsh et al., 2016; Wang et al., 2016). The salivary microbiota is highly diverse and complex (Curtis et al., 2011).

Estrogen and menopause-related hormonal imbalances are believed to affect oral health (Cao et al., 2007). According to the literature (Meurman et al., 2009), together with climacteric complaints, various oral discomforts are reported in menopausal women. The main peri- and postmenopausal symptoms include xerostomia (subjective oral dryness) and/or hyposalivation (Mahesh et al., 2014), which may increase the occurrence of mucosal and dental diseases, such as candidiasis. Few studies have investigated the effects of hormone replacement therapy in such patients (Mahesh et al., 2014; Lago et al., 2015), although the existing results show an improvement in symptoms following such treatment (Mahesh et al., 2014; Lago et al., 2015).

The quantitative and qualitative changes in saliva may alter the regular homeostasis of oral health, subsequently leading to specific changes in the salivary bacterial composition (Nasidze et al., 2009, 2011; Belstrøm et al., 2014). However, recent findings have shown that patients with severe hyposalivation do not differ in their bacterial profiles compared with those with normal salivary flow rates (Belstrom et al., 2016), although the corresponding study did not focus on the evaluation of such differences between menopausal and non-menopausal women.

Because the salivary composition may be influenced by the presence of oral diseases, prescribed medications and general health (Belstrom et al., 2016), researchers must pay attention to the sample size and control for confounding factors when revising the existing literature to confirm the external validity of any quantitative and qualitative changes in saliva related to menopause.

### Periodontal Support

The periodontium is the specialized tissue that both surrounds and supports the teeth. Periodontal disease, which includes gingivitis and periodontitis, is highly prevalent in adults, and disease severity increases with age. This inflammatory disease develops over time with the accumulation of biofilm (dental plaque), bacterial dysbiosis, the formation of periodontal pockets, gum recession, and tissue destruction (including alveolar bone loss), which can ultimately lead to tooth loss (Michaud et al., 2017).

Fluctuating female sexual hormone levels in menopausal women may represent key factors that respond to changes detected in the oral cavity (Dutt et al., 2013). Menopause is accompanied by decreased bone density, which may have implications for oral health such as the risk of enhanced progression of periodontal infections and tooth loss (Hernandez-Vigueras et al., 2016). According to the literature, sex-related hormonal changes may cause the gums to become more susceptible to plaque and create a much higher risk for gingivitis and advanced periodontitis (Suresh and Radfar, 2004).

Periodontitis is a chronic inflammatory process that occurs in response to an increase in Gram-negative bacteria in the biofilm (Ruby and Barbeau, 2002), affecting the tissues that surround and support the teeth. Specific bacterial species, such as *Porphyromonas gingivalis* and *Tannerella forsythensis*, were found to be important in the etiology of periodontitis in postmenopausal women (Brennan et al., 2007). In addition, changes in periodontal status were found to be associated with variations in sex hormone levels (Mascarenhas et al., 2003), and the occurrence of periodontitis was reported to be greater in postmenopausal women who did not receive hormone replacement than in premenopausal women (Haas et al., 2009). Therefore, from a clinical point of view, the roles of sex hormones and hormone therapy in the prevalence of subgingival bacterial infection in peri- and postmenopausal women are of great interest.

In a cohort study that included 106 women aged 50–58 years, hormone replacement therapy led to a decreased number of positive samples showing the periodontal pathogens *P. gingivalis, Prevotella intermedia*, and *T. forsythia* from the subgingival plaque (Tarkkila et al., 2010). Consistent with this result, a previous study found improved periodontal probing depths and tooth mobility in 190 randomized women who received hormone therapy for 1 year (López-Marcos et al., 2005). Conversely, Pilgram et al. (2002) investigated 135 women in a randomized, controlled trial who received estrogen replacement for 3 years and did not find any changes in clinical parameters such as the attachment of teeth or the bone mineral density of the lumbar spine. In mice, estrogen seems to modulate IL-1 production and participate in the resistance of females to disseminating dentoalveolar infections, leading to the enhanced localization of these infections (Youssef and Stashenko, 2017), which draws attention to the potential role of sex-related hormones in the modulation of oral mucosal infections.

Non-conventional treatment approaches for oral infections, with a particular emphasis on dental biofilm-related diseases, have gained attention in recent years. The use of probiotics and prebiotics to improve gastrointestinal health has now led to an interest in using these treatments to control oral diseases (Allaker and Ian Douglas, 2015). However, few studies have focused on recovery of the oral equilibrium by promoting beneficial microbiota. Despite differences in the composition of the gut and oral microbiota, the community types observed in the gut are predictive of the community observed in the mouth and vice versa (Ding and Schloss, 2014). Among other host factors, the oral microbiota serves as an inoculum for the intestine, and the microorganisms that find adequate conditions in the mouth give rise to distinct types of communities in the intestine. Interestingly, oral inoculation with *P. gingivalis* in experimental models leads to a change in the intestinal microbiota, which is a possible mechanism for the establishment of diseases associated with periodontitis, such as cardiovascular diseases (Arimatsu et al., 2014). In this sense, understanding the role of health-associated microorganisms may have utility in the application of these approaches for the prevention and treatment of disease (Sanz et al., 2017).

## The Interaction Between Gut Microbiota and Female Sex Hormones

**FIGURE 1:**
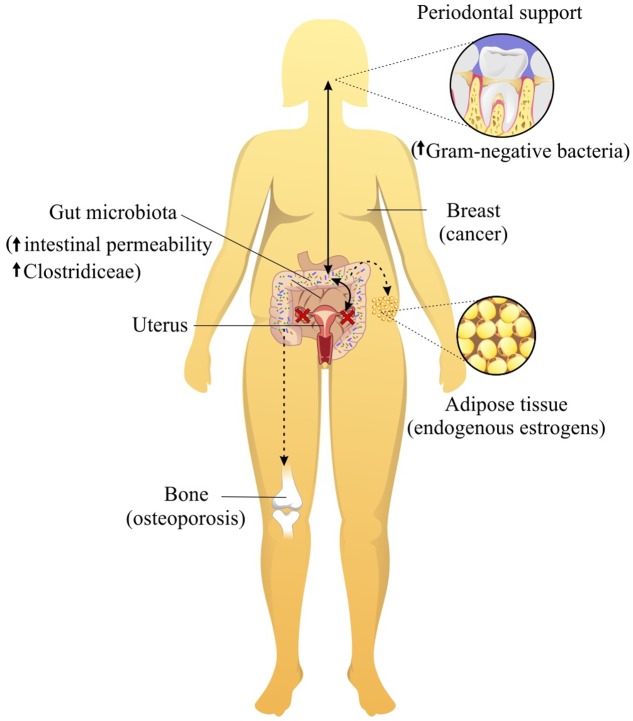
Female sexual hormones levels influence the composition of the microbiota in many sites of the body, especially mouth and gut. The oral and gut microbiota have been shown to influence many diseases, such as osteoporosis, weight gain and lipid deposition, breast cancer and periodontitis.

As mentioned earlier, female sex hormones levels influence the composition of the microbiota in many sites of the body, especially the gut. Due to intimate contact with the larger gut immune system, the gut microbiota has been shown to influence many diseases outside of this organ (**Figure 1**). Accordingly, imbalance of the gut microbiota, called dysbiosis, has been extensively related to metabolic and immunological diseases. Interestingly, the presence or absence of estrogen may be able to alter the gut microbiota equilibrium and corresponding disease pathways. Some autoimmune diseases affect more often women than men, including systemic lupus erythematosus (Jiang et al., 2005), Sjogren’s syndrome (Patel and Shahane, 2014) and rheumatoid arthritis (Oliver and Silman, 2009). Gender differences have also been reported for the outcome of microbial infections (Fischer et al., 2015). Interestingly, the onset of autoimmune diseases, asthma (Akinbami et al., 2016) and other diseases occurs after menarche or during the reproductive period of women. Experimental findings in mice have shown that the interactions among the microbiota, female sexual hormones, and immunity are associated with the development of autoimmune diseases (Yurkovetskiy et al., 2013, 2015), including type 1 diabetes (Markle et al., 2013) and rheumatoid arthritis (Wu et al., 2010). The non-obese diabetic (NOD) mouse exhibits spontaneous, immune-mediated pancreatic beta cell destruction causing type 1 diabetes (T1D) with a complex genetic and environmental etiology. The NOD T1D incidence shows a strong 2:1 female to male sex bias (Markle et al., 2013). Interestingly, germ-free NOD female mice lack this gender bias for diabetes. Additionally, after castration, males exhibit a similar microbiota composition and T1D incidence to females (Markle et al., 2013). In general, this study shows that the microbiome is a causal factor and not simply a consequence of autoimmune disease.

### The Relevance of the Gut Microbiota in the Health of Menopausal Women

When the interaction between the gut microbiota and estrogen is altered due to a lack of estrogen, this relationship is restructured according to the new circumstances. However, host functional alterations, such as metabolic and immunological changes, also occur.

Obesity affects 65% of postmenopausal women and is associated with the onset of metabolic dysfunction (Leeners et al., 2017). Multiple studies have suggested that postmenopausal women exhibit increased total fat mass and abdominal fat and decreased lean body mass compared with those of premenopausal women, regardless of aging (Aloia et al., 1995; Schreiner et al., 1996; Cordina-Duverger et al., 2016). The accumulation of abdominal fat in postmenopausal women appears to be a critical factor in the development of insulin resistance and type 2 diabetes (Lobo et al., 2014), and the relationship between the gut microbiota and a lack of estrogen is likely responsible for weight gain and lipid deposition during menopause (**Figure 1**). The gut microbiota can metabolize estrogen-like compounds such as isoflavonoids, which are found in soy foods, and promote the growth of some specific bacteria (Frankenfeld et al., 2014; Chen and Madak-Erdogan, 2016; Miller et al., 2017). Indeed, the administration of soy isoflavones to postmenopausal women was shown to increase the concentration of *Bifidobacterium* and suppress Clostridiaceae, which are known to be involved in inflammatory diseases (Frankenfeld et al., 2014; Nakatsu et al., 2014). This suppression of Clostridiaceae, a family of Clostridia associated with obesity (**Figure 1**), likely explains why diets containing phytoestrogens have been shown to improve weight gain in menopausal women.

Few studies have investigated whether prebiotics and probiotics can improve insulin sensitivity in postmenopausal women or body fat in mice. The intake of flaxseed mucilage, a prebiotic, is known to improve insulin sensitivity and alter the gut microbiota in obese postmenopausal women (Brahe et al., 2015). Thus far, the implications of the gut microbiota with low levels or the absence of estrogen hormone in the metabolism of women have not been sufficiently studied and require further clarification.

Another link between the gut microbiome and menopausal health is related to bone. Interesting, the gut microbiota has also been found to influence bone homeostasis. Approximately one in two women over age 50 will break a bone because of osteoporosis. A study that involved twenty postmenopausal women with a mean age of 65 years showed that the group that consumed *Lactobacillus helveticus*-fermented milk had increased serum calcium levels and reduced bone reabsorption compared with those of the control milk consumption group (Narva et al., 2004). Experimental studies have also demonstrated similar results. For instance, *L. reuteri* treatment significantly protected ovariectomized mice from bone loss and increases in bone marrow CD4+ T-lymphocytes, which promote osteoclastogenesis (Britton et al., 2014). Another study that investigated probiotic treatment for cortical bone loss found reduced expression of two inflammatory cytokines, TNF-α and IL-1β, and increased expression of osteoprotegerin, a potent inhibitor of osteoclastogenesis, in the cortical bone of ovariectomized mice (Ohlsson et al., 2014). Additionally, sex steroid deprivation has been reported to promote intestinal permeability (**Figure 1**), and the oral administration of *L. rhamnosus* GG (LGG) or VSL#3 (a combination of tree probiotics) to estrogen-deficient mice significantly reinforced intestinal barrier integrity and completely protected the mice against sex steroid depletion-induced bone loss (Li et al., 2016). Importantly, to confirm the role of the gut microbiota in bone health, another experiment also showed that germ-free mice are protected against the bone loss induced by the absence of sex steroids (Li et al., 2016).

We must mention that the gut microbiota may influence the risk for breast cancer through effects on endogenous estrogens produced by adipose tissue in postmenopausal women (**Figure 1**) (Key et al., 2003). A cross-sectional study on 60 healthy postmenopausal women found that women with a more diverse gut microbiome and an abundance of four Clostridia taxa exhibited an elevated urinary ratio of hydroxylated estrogen metabolites to parent estrogens (Fuhrman et al., 2014), which is related to the etiology of breast cancer (Flores et al., 2012; Kwa et al., 2016). However, another study compared 48 postmenopausal breast cancer patients and 48 control patients and observed that postmenopausal women with breast cancer exhibited an altered composition and estrogen-independent low diversity of their microbiota (Goedert et al., 2015). These different findings on gut microbiota diversity and breast cancer could be explained by the fact that disease outcome or disease stage can also affect the microbiota. In this scenario, the consumption of the soy isoflavone daidzein, which is metabolized by some bacteria of the microbiota to generate equol and *O*-desmethylangolensin (ODMA), could represent a therapeutic strategy for breast cancer prevention. Some, but not all, studies have shown a lower risk of breast cancer associated with equol production (Hullar et al., 2014). However, only approximately 30–50% of the population can metabolize daidzein (Frankenfeld et al., 2004; Uehara, 2013; Nakatsu et al., 2014) to equol, likely due to the host microbiota. Therefore, an investigation into the administration of the soy isoflavone daidzein together with probiotic bacteria to produce equol is warranted and could offer benefits in the prevention of breast cancer in menopausal women.

## Concluding Remarks

Many chronic diseases can emerge after estrogen levels decline, which will affect a considerable part of a woman’s life. Understanding the role of the microbiota in women’s health at the menopausal phase could help to improve strategies for microbiota modulation and prevent dysfunction. The oral and gut microbiotas have been extensively studied in women of reproductive age, while the menopausal period has been somewhat overlooked. The use of hormone replacement is not indicated for all menopausal women, and considering that probiotics and prebiotics can affect the dysfunction of bone, adipose tissue, oral and other tissues, such treatments may constitute an important therapeutic strategy. Pro- and prebiotics can also be used in conjunction with menopause hormone therapy and may attenuate the side effects that can arise from hormone replacement. In conclusion, the scientific findings published to date do not definitively demonstrate how non-vaginal microbiota sites influence the health of menopausal women. Thus, many questions remain unanswered and warrant further investigation to improve the quality of life of menopausal women.

## Author Contributions

AV, PC, DR, and CF drafted and revised the manuscript.

## Conflict of Interest Statement

The authors declare that the research was conducted in the absence of any commercial or financial relationships that could be construed as a potential conflict of interest.
